# North Atlantic storm track changes during the Last Glacial Maximum recorded by Alpine speleothems

**DOI:** 10.1038/ncomms7344

**Published:** 2015-02-27

**Authors:** Marc Luetscher, R. Boch, H. Sodemann, C. Spötl, H. Cheng, R. L. Edwards, S. Frisia, F. Hof, W. Müller

**Affiliations:** 1 Institute of Geology, University of Innsbruck, Innsbruck 6020, Austria; 2 Swiss Institute of Speleology and Karst Studies—SISKA, 2301 La Chaux-de-Fonds, Switzerland; 3 Institute of Applied Geosciences, Graz University of Technology, 8010 Graz, Austria; 4 Institute for Atmospheric and Climate Science, ETH, 8092 Zurich, Switzerland; 5 Geophysical Institute, University of Bergen, 5020 Bergen, Norway; 6 Institute of Global Environmental Change, Xi'an Jiaotong University, Xi'an 710049, China; 7 Department of Earth Sciences, University of Minnesota, Minneapolis, 55455 Minnesota, USA; 8 School of Environmental and Life Sciences, University of Newcastle, Callaghan, NSW 2308, Australia; 9 Swiss Society of Speleology, 2301 La Chaux-de-Fonds, Switzerland; 10 Department of Earth Sciences, Royal Holloway University of London, Egham TW20 0EX, UK

## Abstract

The European Alps are an effective barrier for meridional moisture transport and are thus uniquely placed to record shifts in the North Atlantic storm track pattern associated with the waxing and waning of Late-Pleistocene Northern Hemisphere ice sheets. The lack of well-dated terrestrial proxy records spanning this time period, however, renders the reconstruction of past atmospheric patterns difficult. Here we present a precisely dated, continuous terrestrial record of meteoric precipitation in Europe between 30 and 14.7 ka. In contrast to present-day conditions, our speleothem data provide strong evidence for preferential advection of moisture from the South across the Alps supporting a southward shift of the storm track during the local Last Glacial Maximum (that is, 26.5–23.5 ka). Moreover, our age control indicates that this circulation pattern preceded the Northern Hemisphere precession maximum by ~3 ka, suggesting that obliquity may have played a considerable role in the Alpine ice aggradation.

## Abstract (web summary)

Insights into Late-Pleistocene Northern Hemisphere storm track variability are hampered by a lack of well-dated proxy records. Here, the authors present a precisely dated record of meteoric precipitation between 30 and 14.7 ka, and show that obliquity may have played a vital role in Alpine glacier advance.

## Graphical abstract

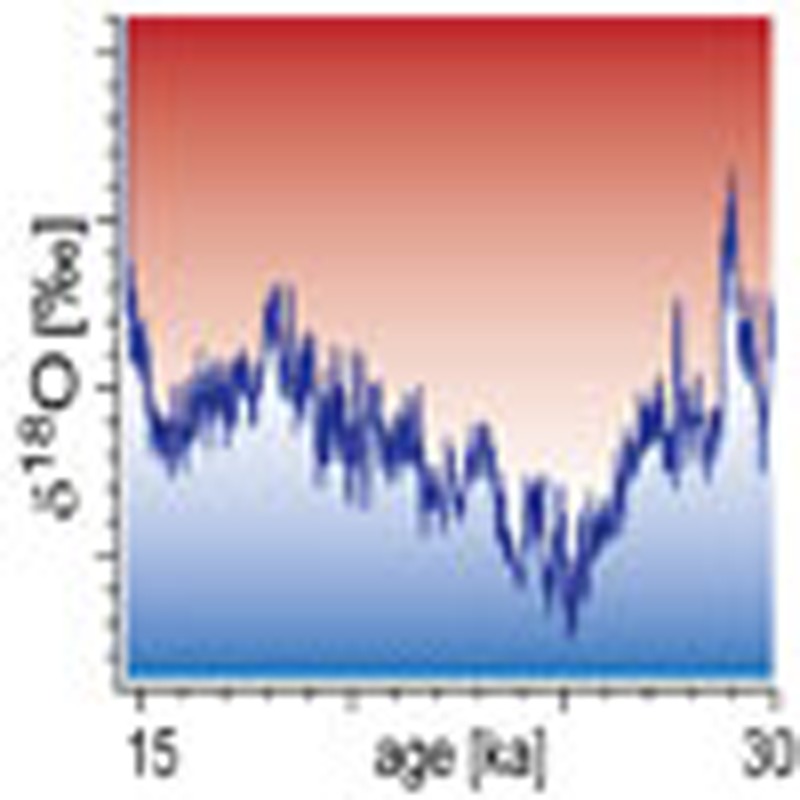

The Last Glacial Maximum (LGM) characterizes the climax of a pronounced sea-level minimum associated with the global ice extent during the Upper Pleistocene, between ca 26.5 and 19 ka (refs 1, 2). While atmospheric circulation models support a southward shift of the North Atlantic storm tracks to ~40 °N (refs 3, 4) paleoclimate proxy records point to a global mean temperature decrease of between 4 and 7 °C when compared with preindustrial times^5^. In the European Alps, the extent of the regional ice sheet has been comprehensively documented by detailed geomorphological mapping^6^,^7^. Observations of trimlines reveal the presence of three major ice domes, all located south of the present climate divide, suggesting that precipitation was mostly associated with air advection from the South^8^. However, in the absence of continuous terrestrial palaeoclimate records from the northern Alpine mountain chain, it is yet unclear whether this precipitation pattern was associated with increased cyclogenesis in the western Mediterranean^9^ or if it relates to an overall shift in the storm trajectories from the eastern North Atlantic^8^. This uncertainty in regional atmospheric circulation patterns has major implications, primarily for the regional ice build-up and thus the timing of valley glacier advances but also for the distribution of temperature and precipitation responses and thus implicitly also for vegetation refugia.

Reconstructing LGM atmospheric circulation in the European Alps remains inherently difficult and requires accurately dated high-resolution climate proxy records. High-altitude ice cores certainly represent the most direct precipitation archive at mid-latitudes, but barely cover the Holocene^10^. Ice and permafrost conditions in the Alpine foreland further hinder formation/preservation of possible lake records and biogenic proxies during the LGM. Speleothems, secondary carbonate deposits found in caves, represent an alternative archive for Late Pleistocene climate history provided that the temperature in the subsurface stayed above freezing and local water–rock interactions gave rise to calcite-supersaturated water.

Here we present the first continuous continental European record of meteoric precipitation obtained from speleothems grown during Marine Isotope Stage 2. We show that our record reflects atmospheric circulation patterns in the Northern Hemisphere during the LGM, supporting a southward shift of the North Atlantic storm track about 3 ka before the precession maximum. We conclude that the preferential advection of moisture from the South likely favoured regional differences in the ice build-up in the Alps, which suggests contrasting responses of valley glacier advances during the LGM.

## Results

### Study site

**Figure 1:**
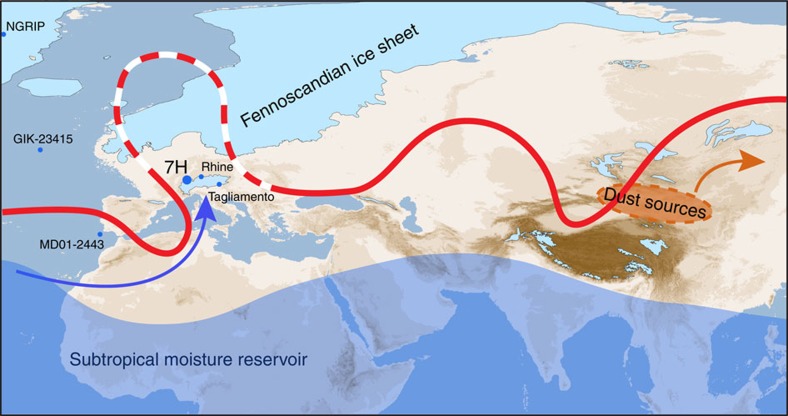
Sieben Hengste site location. Conceptual view of southerly moisture transport to the Alps during the Last Glacial Maximum (blue arrow) from the subtropical reservoir (blue shading). The advection of moisture is triggered by Rossby-wave breaking of the jet stream over Western Europe (red line), which is enhanced due to the presence of the Fennoscandian ice shield (light blue; *6*) and the associated semi-permanent blocking high. Dust-mobilizing storms are induced further East by jet stream excursions propagating to Central and Eastern Asia (orange area and arrow). 7H: Sieben Hengste cave system; NGRIP: ice core; GIK-23415 and MD01-2443: deep sea sediment cores; Rhine and Tagliamento: palaeoglaciers.

Two coeval speleothems (7H-2 and 7H-3) were sampled in the Sieben Hengste (7H) cave system (Bernese Alps, Switzerland; Fig. 1), a network comprising more than 150 km of explored cave passages. The galleries formed within Urgonian carbonates (Schrattenkalk Formation), which are locally overlain by low-permeable Eocene sandstones (Supplementary Fig. 1). Several lines of evidence, including alignments of erratic boulders and glacial striations, point to an ice-covered hydrological catchment at 1,700–2,000 m a.s.l. during the LGM^7^. Based on geomorphological evidence from the nearby Napf palaeoglacier, the local Equilibrium Line Altitude (ELA) during the LGM is estimated at ca 1,250 m a.s.l. Despite the low temperatures expected during the LGM, summer rainfall and seasonal melting of winter-snow must have been sufficient to allow for recharge into the Sieben Hengste karst system and ensure speleothem deposition. Concentrated infiltration along major tectonic discontinuities likely maintained a locally constant temperature boundary condition in the epikarst close to the melting point (that is, 0 °C), whereas hydrological drainage ensured geothermal heat advection off the phreatic zone. Accordingly, the vertical temperature distribution in the Sieben Hengste karst system most likely followed the adiabatic lapse rate of humid air^11^, implying an isotherm of ca 1.0±0.5 °C at 1,500 m a.s.l.

### Speleothem samples

The two stalagmites (Supplementary Fig. 2) were collected ca 215 m below the ground surface (that is, 1,540 m a.s.l.) in an alcove at the base of a vadose shaft. The candle-shaped stalagmites, 57- and 30-cm long show similar petrographies dominated by bundles of elongated columnar fascicular optic calcite crystals, commonly arranged in fans (Supplementary Fig. 3). This fabric is typical of elevated Mg/Ca ratios in the drip water and agrees with the chemical concentrations measured in the speleothem (Supplementary Table 1). Both, the calcite fabrics and the regular stalagmite morphologies suggest that the karst aquifer properties remained constant over decadal to millennial timescales. The regular recharge was mainly controlled by the glacier hydrology. Hence, the temperature in the cave remained largely constant, and we surmise that the 7H δ^18^O is primarily controlled by changes in the precipitation isotope composition.

### The 7H-record

The 50 uranium-series ages obtained by multicollector inductively coupled plasma mass spectrometry are all in stratigraphic order and reveal continuous calcite deposition from 30.0 to 14.7 ka b2k (Supplementary Table 2). Typical age uncertainties of less than 0.5% reflect the elevated ^238^U concentration of 1,200±300 ng g^−1^ associated with low detrital thorium contents. The age model reveals an average growth rate of 40–70 μm a^−1^ (Supplementary Fig. 4). Sub-samples for δ^18^O and δ^13^C analyses were milled at 200 μm intervals along the speleothem growth axis providing an average resolution of 4.5 a.

**Figure 2:**
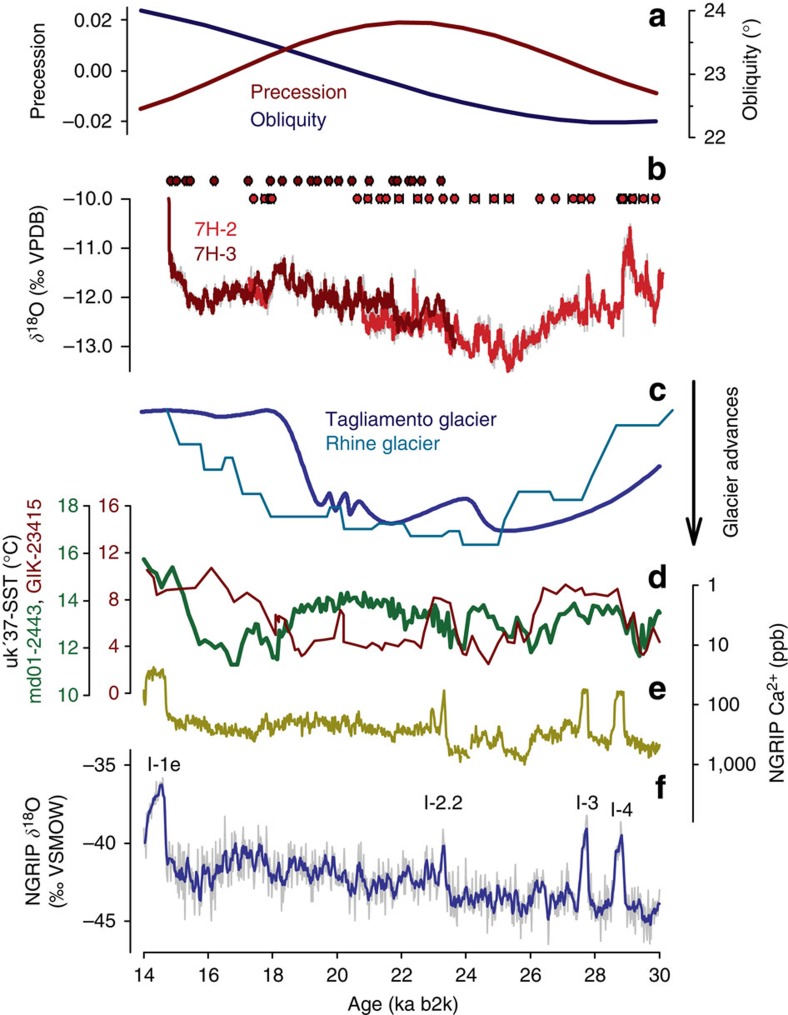
LGM data comparison. (**a**) Precession (brown) and obliquity (blue)^23^. (**b**) 7H-2 (red) and 7H-3 (brown) δ^18^O time series (21a low-pass filter) plotted with individual U/Th ages and measured errors (2σ). (**c**) Alpine glacier advances reconstructed for Tagliamento and Rhine glaciers^17^,^18^. (**d**) North-eastern Atlantic sea-surface temperature^15^,^16^. (**e**) NGRIP dust record^33^. (**f**) NGRIP δ^18^O with a 100a low-pass filter^12^.

The speleothem oxygen isotope composition (ca 4,200 analyses) ranges between δ^18^O_VPDB_ −10.5 and −13.5‰ and oscillates on decadal to millennial time scales (Fig. 2b). The stalagmite δ^18^O time series correlate in overlapping segments and, therefore, likely responded to the same hydrological forcing. This hydrological control is emphasized by a high local correlation (*r*^2^=0.54) between δ^18^O and δ^13^C, which suggests that any decrease in drip rate related to lower recharge forced a coherent C isotope change (Supplementary Fig. 5). Overall, δ^13^C values stayed remarkably constant at +4.4±0.4‰, indicating that the dissolved inorganic carbon was largely derived from the host rock. A composite δ^18^O record was obtained by using a Monte Carlo (MC) approach to find the best-possible correlation between adjacent time series (see Methods for details).

## Discussion

The 7H δ^18^O record shows remarkable similarities to Northern Hemisphere palaeoclimate records, both at orbital and millennial timescales. Positive δ^18^O excursions of 7H (Fig. 2b) match the North Greenland Ice Core Project (NGRIP) δ^18^O record (Fig. 2f) for Interstadials (I) 3 and 4 within dating uncertainties^12^,^13^ (Table 1). The timing of the rapid δ^18^O increase at the onset of the Bølling (that is, I-1e at 14.70 ka) is identical with the timing in NGRIP and led to a stalagmite growth stop due to the waning of the glacier cover in the hydrological catchment of the cave system. We argue that the high degree of similarity between the 7H-record and Greenland ice cores reflects the common variability of the North Atlantic climate system^14^. This interpretation is supported by coeval changes in the sea-surface temperature recorded off the Iberian Peninsula^15^ (core 2443 in Fig. 2d). This covariance is highest when the near-surface poleward heat transfer, between 26.3 and 23.5 ka, was strongly reduced^16^(for example, core 23415 for comparison in Fig. 2d), suggesting internal feedback mechanisms associated with variations in the Atlantic Meridional Overturning Circulation.

In contrast to the NGRIP record, the 7H δ^18^O record is characterized by an absolute minimum centred at ca 25.3 ka, consistent with the maximum Alpine glacier advance inferred from independently dated geomorphological field evidence^17^,^18^ (Fig. 2c). In addition, second-order changes in the 7H δ^18^O time-series correlate within dating uncertainty with minor glacier oscillations reconstructed from proglacial sediments^17^ and agree with a perialpine vegetation record from Les Echets, eastern France^19^, which has been interpreted to reflect primary changes in soil moisture availability. Based on these evidences, we surmise that the 7H δ^18^O record mirrors changes in the mass balance of LGM glaciers in the Alps and thus predominantly reflects changes in the Alpine precipitation pattern^8^.

The modern regime of moisture transport to Sieben Hengste is dominated by westerly advection (Supplementary Fig. 6), while about 10–30% of the annual precipitation is transported on average from the South across the Alpine mountain range. Intense southerly moisture advection is known to be related to pronounced Rossby-wave breaking west of the Alps, which induces enhanced meridional flow of moist and warm subtropical air towards the mountain range^20^. Such transport patterns may have been dominant during the LGM when both a larger (winter) sea-ice cover in the North Atlantic^21^ displaced the baroclinic zone further south and a semi-persistent blocking high over the Fennoscandian ice sheet deflected the North Atlantic jet stream substantially, leading to more frequent Rossby-wave breaking (Fig. 1). Since the tropics and subtropics experienced comparatively less temperature changes during glacial-interglacial cycles, the subtropical moisture reservoir could have provided sufficient moisture for precipitation to build up and maintain ice domes in the Alps with meridional moisture transport events. The strong correlation between the 7H-record and the Alpine ice mass balance (Fig. 2c) suggests that this atmospheric circulation pattern may have been a characteristic mechanism of moisture delivery to the Alps during the LGM.

**Figure 3:**
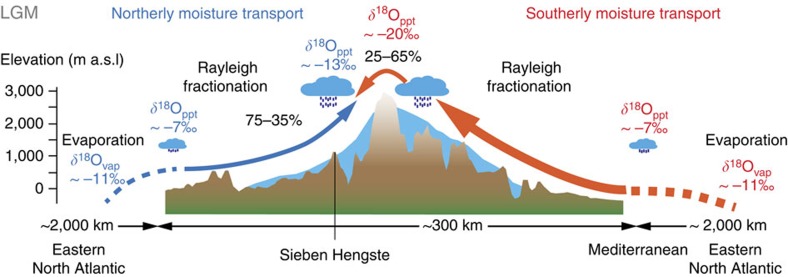
Conceptual model of isotope fractionation in meteoric precipitation along two distinct trajectories. Rayleigh isotope fractionation along the northwestern (blue) and southern moisture transport trajectory (red) for the Last Glacial Maximum. Moisture evaporates from the source region in the Eastern North Atlantic and then follows a fractionation path with orographic rainout up to 1,700 m a.s.l. during northerly advection, or up to 3,000 m a.s.l. for the southerly advection across the Alpine main crest. δ^18^O values are expressed in VSMOW. Percentages reflect the proportion of precipitation reaching 7H along the northwestern and southern routes, respectively. The ranges reflect uncertainties on the interannual variability (see Supplementary Discussion for details). Based on the 7H δ^18^O record, we surmise that the proportion of North Atlantic moisture transported from the South to 7H during the LGM was two to three times higher than today.

**Figure 4:**
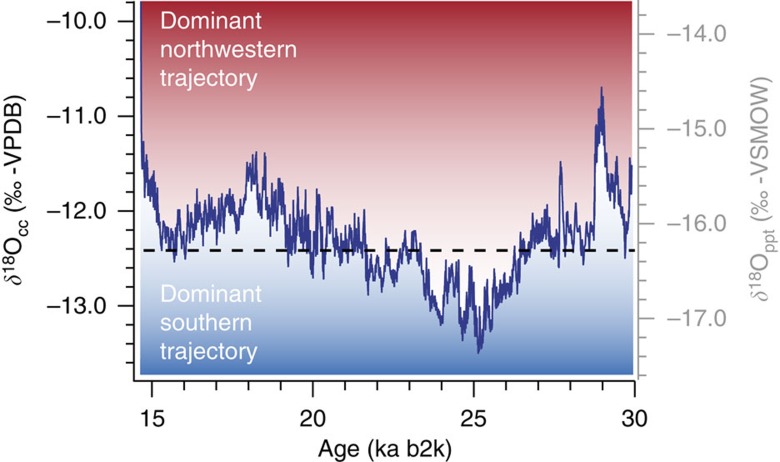
The composite 7H δ>^18^O record. The correlation with the Alpine Tagliamento glacier advances^17^ (Fig. 2c) suggests that low δ^18^O values are primarily associated with moisture transport from the South. The stippled line marks the approximate equilibrium between northwestern and southern moisture transport as identified from the Remanzacco oscillations. A dominant northwestern trajectory reflects a more northerly storm track position, and vice-versa. The secondary *y* axis shows the inferred isotopic composition of meteoric precipitation assuming calcite precipitation close to thermodynamic equilibrium^34^ in a cave environment just above freezing point.

Accordingly, we propose that, during the LGM, the isotopic composition of 7H primarily recorded changes in the position of the North Atlantic storm track and associated changes of moisture advection to the Alps. This hypothesis is generally consistent with global climate simulations suggesting an overall southward displacement of the storm track during the LGM^3^,^4^, accompanied by a significant precipitation increase in the mid-latitudes, in particular around the Iberian Peninsula^21^. Meanwhile, reconstructed meridional temperature gradients based on Alpine glacier’s ELA depression suggest cold and dry conditions in the northern Alps at the LGM climax^22^. The presence of liquid water in the 7H karst system at 1,750 m a.s.l., however, challenges an estimated mean annual air temperature of −10 to −15 °C at the ELA of glaciers^22^ and suggests that major precipitation events must have been associated with substantial advection of warm moist air, predominantly between spring and autumn. Owing to their transient nature, such events are hardly reflected in the mean annual air temperature record^23^ and the relatively small changes in the temperature of condensation only had a minor influence on the isotopic composition. Similarly, the extraordinarily cold and dry climate during the LGM makes it unlikely to explain the depletion in δ^18^O_cc_ by a seasonal shift towards more winter precipitation. Instead, we propose that a higher fraction of precipitation was transported across the Alpine mountain range from the South because of a change in the predominant synoptic circulation pattern. The higher orographic barrier along the southerly transport path cause more moisture to condensate resulting in an orographically induced Rayleigh fractionation process (Supplementary Fig. 7). Accordingly, phases of more depleted δ^18^O_cc_ are associated with the preferential advection of moisture from the South (see Supplementary Discussion), whereas less depleted δ^18^O_cc_ values primarily reflect air masses reaching the northern Alps directly from the Northwest (Fig. 3). Based on the apparent correlation between the Remanzacco oscillations of the LGM South Alpine Tagliamento paleoglacier^17^ and the 7H-record, the 50% threshold is assessed at δ^18^O_cc_ −12.4±0.1‰, which corresponds to ca −16.3‰ on the Vienna Standard Mean Ocean Water (VSMOW) scale (Fig. 4). Because δ^18^O at the oceanic moisture source has not changed substantially during the LGM, a two end-member mixing model allows to derive that the contribution of moisture crossing the Alps may have varied between 25 and 65% during the LGM.

Associating the (Alpine) LGM with a change in the synoptic circulation pattern rather than an insolation minimum enables alternative interpretations for the causes of major glacier advances in the Alps. We note that the 7H δ^18^O minimum predated the precession maximum at 22 ka (ref. 24) by ca 3 ka, suggesting a combined effect of precession and obliquity on the timing of the last Alpine glaciation, and thus possibly also on its global counterpart^25^. Evidence of de-phasing with respect to the precessional band has been reported from marine and terrestrial records^26^, but interpretations were mainly focused on the timing of deglaciation. Although changes in obliquity influence the latitudinal distribution of solar radiation and thus control glacier ablation^27^, its effect on ice accumulation is less obvious. Yet, the obliquity minimum at 29 ka (ref. 24) implies reduced energy input at high latitudes. The associated cooling of the Northern Hemisphere likely favoured a drop in the ELA, as exemplified by the start of speleothem growth at 7H at 30 ka. With tropical and subtropical areas being less affected by changes in obliquity, meridional temperature gradients and therefore also pressure gradients became steeper, strengthening the jet stream, which, together with an expansion of the Fennoscandian ice sheet, progressively migrated southwards^3^. This may have led to enhanced synoptic storm activity in North-Atlantic mid-latitudes, and possibly also in areas further downstream, because of jet stream excursions induced by the interaction between the jet and a quasi-stationary blocking over the Fennoscandian ice sheet (Fig. 1). The concomitant decline of the mid-latitude vegetation cover increased the potential global dust emissions by nearly 38% (ref. 28). An intriguing similarity is noted between the Ca record of NGRIP and the 7H δ^18^O record (Fig. 2e), which is particularly pronounced between 28 and 23 ka (that is, *r*≥0.60 between 25.5 and 23.0 ka; Supplementary Fig. 8). Elevated dust concentrations in Greenland ice cores correlate with depleted δ^18^O values in 7H. This increased dust mobilization during periods when the storm track was dominantly south of the Alps supports a potential teleconnection between North Atlantic moisture transport and storminess in East Asian regions (Supplementary Discussion). Concurrently, the preferential transport of moisture towards the southern flank of the Alps favoured snow accumulation in the glaciological catchment of the large piedmont glaciers. These results are not only consistent with independent geomorphological evidence^7^,^8^ but also argue for a non-uniform ice build-up in the Alps during the LGM. Accordingly, we expect regional differences in the timing of valley glacier advances depending on the location of the glaciers’ accumulation areas.

We conclude that the Alpine glaciers responded in a sensitive way to Northern Hemisphere atmospheric circulation patterns during the LGM. Therefore, this mountain range represents a key location for identifying large-scale atmospheric reorganizations in the North Atlantic climate systems. The importance of moisture advection towards the Alps for glacier advances calls for high-resolution GCM simulations of the LGM climate, which could be tested extensively using 7H as a benchmark.

## Methods

### U/Th dating and age modelling

Chemical separation and MC-ICP-MS (Thermo-Finnigan Neptune) measurements of U and Th isotopic ratios were undertaken at the University of Minnesota using procedures similar to those described in ref. 29. The extent of detrital ^230^Th contamination was estimated and corrected for by measurement of the long-lived chemically equivalent ^232^Th and assuming a silicate bulk Earth initial ^230^Th/^232^Th atomic ratio of 4.4±2.2 × 10^−6^. Final ages are given as years before the year 2000 AD (a b2k). Age modelling was performed using StalAge^7^, an algorithm specifically designed for speleothems.

### Speleothem geochemistry

Trace elements were measured by Laser ablation inductively coupled plasma mass spectrometry (LA-ICPMS) (RESOlution M-50 prototype at RHUL, 193 nm, ArF excimer) with a two-volume LA cell coupled to an Agilent 7500ce quadrupole ICPMS^30^ along a ca 8-cm-long profile at the base of the stalagmite (single track). Reported concentrations were calculated following ref. 31 using NIST610 as bracketing external standard and stoichiometric [Ca] of 40% m/m as internal standard.

Sub-samples for δ^18^O and δ^13^C analyses were milled at 200 μm intervals along the vertical growth axes of the stalagmites using a Merchantek micromill. Analyses of 0.05–0.35 mg calcite powders were performed at the University of Innsbruck on a ThermoFisher Delta^plus^XL isotope ratio mass spectrometer with an analytical precision (1σ) of 0.08‰ for δ^18^O and 0.06‰ for δ^13^C. Results are reported on the Vienna Pee Dee Belemnite (VPDB) scale and calibrated against NBS19.

### The composite 7H-record

The composite 7H δ^18^O record (Fig. 4) was obtained using a MC approach applied on absolute age determinations to find the best correlation between adjacent time series (ISCAM^32^). We prescribed a point-wise linear interpolation between data points. The age model was determined from the highest correlation obtained over 100,000 MC simulations using a 50a smoothing. The significance estimation was performed using 2,000 pairs of artificially constructed first-order autoregressive time series (AR1), while each pair was scanned with 1,000 MC simulations to find the best correlation. Finally, the result was re-sampled at annual intervals, detrended and normalized to be compared with other proxy records.

## 

## Author contributions

M.L. designed the study, completed the field work together with F.H. and interpreted the results. R.B. carried out the U/Th analyses with support of H.C. and R.L.E. H.S. provided the moisture source analysis; C.S. generated the stable isotope data and contributed to the interpretation together with H.S., S.F. helped in the analysis and interpretation of the speleothem petrography and W.M. carried out the trace element analyses. M.L. wrote the manuscript and designed the figures with input from all co-authors.

## Additional information

**How to cite this article:** Luetscher, M. *et al.* North Atlantic storm track changes during the Last Glacial Maximum recorded by Alpine speleothems. *Nat. Commun.* 6:6344 doi: 10.1038/ncomms7344 (2015).

## Supplementary Material

Supplementary Information Supplementary Figures 1-8, Supplementary Tables 1-2, Supplementary Discussion and Supplementary References

## Figures and Tables

**Table 1:** Comparison of event boundaries as determined in Greenland and their counterparts in 7H.

	**Greenland**^13^		**7H**	
	**Age b2k**	**Maximum counting error**	**U/Th age b2k**	**Error 2σ**
I-1e	14 692	186	14 708	±40
S-2.1	22 900	573	22 809	±47
I-2.1	23 020	583	22 938	±50
S-2.2	23 220	590	23 021	±46
I-2.2	23 340	596	23 280	±37
S-3	27 540	822	27 686	±65
I-3	27 780	832	27 854	±54
S-4	28 600	887	28 706	±64
I-4	28 900	898	29 109	±66

An event is defined by the first clear signal deviating from the base line before the transition^13^.
