# Supplementary material for: North Atlantic storm track changes during the Last Glacial Maximum recorded by Alpine speleothems
Source: Nat Commun. 2015 Feb 27;6:6344. doi: 10.1038/ncomms7344 (PMC4351561; doi:10.1038/ncomms7344)
Supplement: Supplementary Information — Supplementary Figures 1-8, Supplementary Tables 1-2, Supplementary Discussion and Supplementary References [file ncomms7344-s1.pdf]

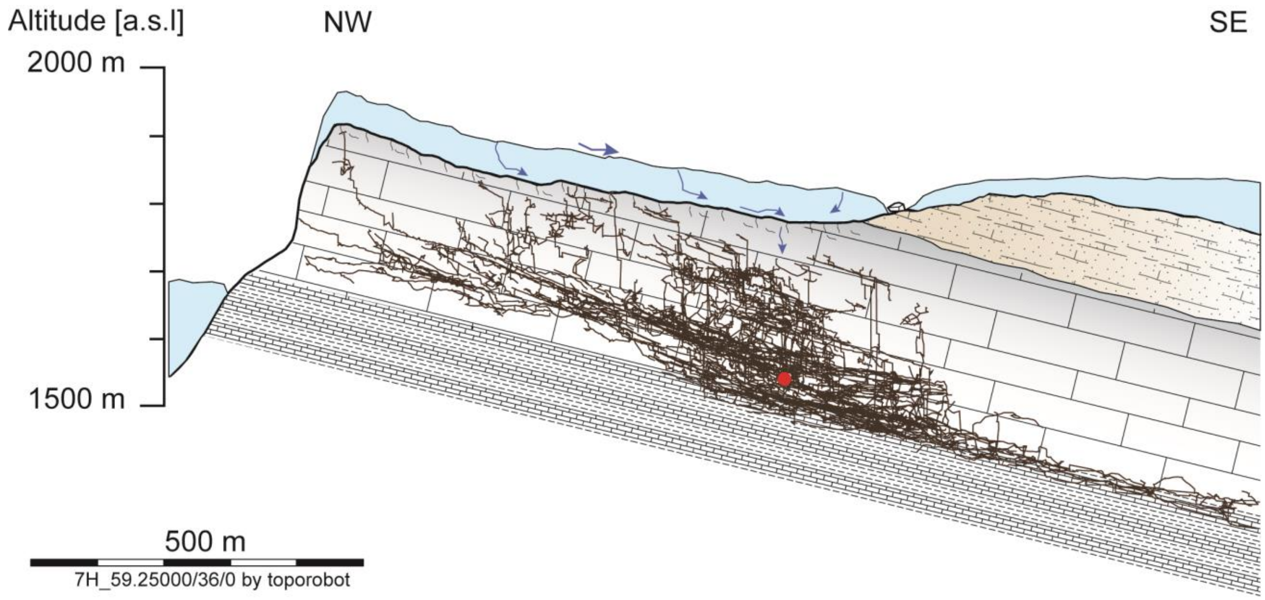

Supplementary Figure 1 The Sieben Hengste karst system during the Last Glacial Maximum

The Sieben Hengste cave network (here on a vertical cross section) is a complex multiphased speleological system (*1*) located north of Lake Thun, Switzerland. The network comprises more than 157 km of surveyed cave passages, expanding over a surface area of ca. 22 km^2 for a vertical range of 1340 m. The karst aquifer encompasses the 200 m-thick Schrattenkalk Formation (Barremian to Aptian; Urgonian facies, grey shading), a Cretaceous platform limestone formed in the Helvetic realm (*2*). The stratigraphic sequence includes a succession of neritic carbonates comprising chiefly oolithic limestones with variable proportions of dolomite. Bioclasts are mainly present as cm-sized rudists but benthic forams, gastropods and echinodea are also observed (*3*). The geological structure follows a monoclinal slope dipping to the southeast at about 15-30° and is delineated to the east by a large normal fault whose throw reaches up to 1000 m. Low-permeable Eocene sandstones (brown shading) cover part of the surface area above the cave. Between 1700 and 2000 m a.s.l., however, the hydrological catchment chiefly comprises denudated Schrattenkalk characterized by limestone pavements. Erratic boulders and glacial polish point to an ice-covered catchment during the Last Glacial Maximum (blue). Based on observations in the nearby Napf area (4), the equilibrium line altitude during the maximal glacier advance is estimated at ca. 1250 m a.s.l. The LGM-speleothems were sampled in a small alcove at the base of an active vadose shaft, ca. 215 m below the modern ground surface (red dot). The present-day aquifer recharge occurs predominantly during snow melt in spring and during summer storm events. Surface runoff is typically absent in the catchment, but active sinkholes form at the contact with the sandstone draining substantial amounts of water into the underneath karst system. The measured mean annual cave air temperature in this part of the cave system is 4.2±0.1°C and active water infiltration is being observed throughout the year. Modern calcite precipitates from various parts of the cave system yield $\delta^{18}O$ values of -7±1 ‰.

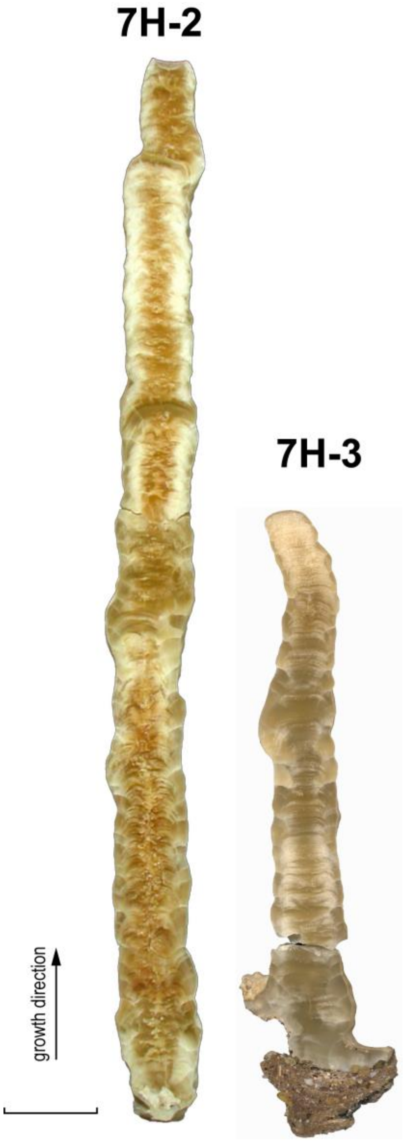

Supplementary Figure 2 The 7H LGM-speleothems

The two stalagmites were found broken on a collapsed sediment bank. The samples show a similar candle-shaped morphology, which suggests a constant drip rate throughout the period of formation. The absence of significant morphological changes suggests that the mean saturation index (Sicc) must have remained constant throughout the growth history (ca. 0.35 to 0.45 for discharge rates of ca. 0.1 l/day; *5*). The 568 mm-long 7H-2 stalagmite, ca 40 mm in diameter, is honey-coloured. Corrosion features are observed on the outer surface, including a small pool at the drip impact on top of the stalagmite. Approximately 57 mm distance from top (DFT), the growth axis shifted by ca. 10 mm. Age modelling (StalAge; *6*) based on 29 multi-collector MC-ICP-MS U/Th ages associates this shift with a ca. 3ka hiatus but, otherwise, reveals a regular deposition between 30 and 17.2 ka with an average growth rate of 66±27 μm a^{-1} (Supplementary Figure 4). 7H-3 is 302 mm long and shows very similar features to 7H-2 (shape, colour, corrosion patterns). A shift in the growth axis is observed 43 mm DFT likely associated with the subsidence of the fine-grained sediment on which it grew. U/Th ages reveal a very regular growth of 39±11μm a^{-1}, between 22.9 and 14.6 ka, and no sign of a hiatus. Scale bar: 50 mm.

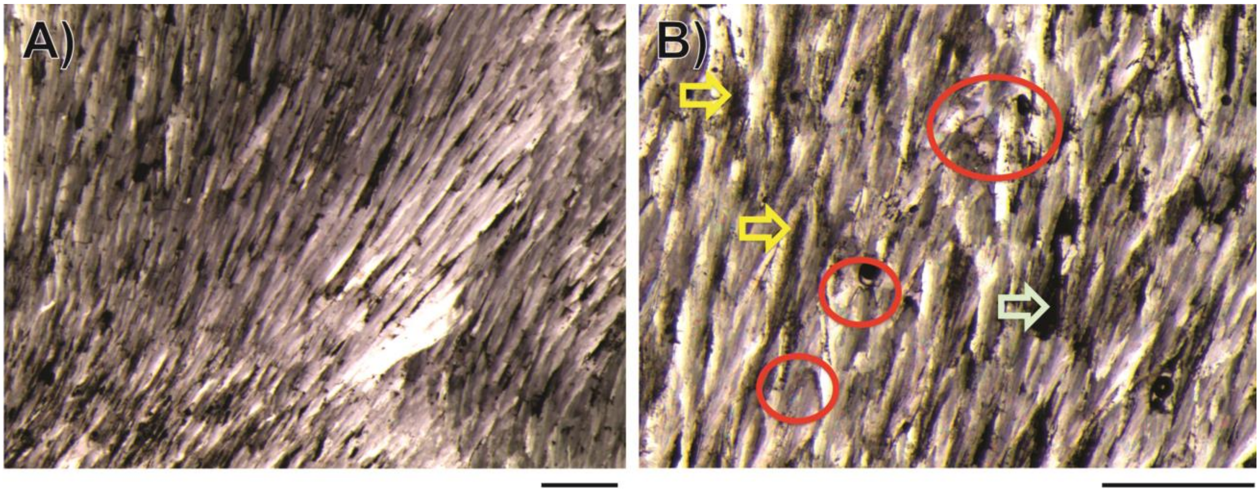

Supplementary Figure 3 Thin section photomicrographs of 7H stalagmites under crossed polars

The stalagmite petrography is characterized by two types of fabrics comprising bundles of compact and more porous fascicular-optic calcite crystals showing typically divergent optic axes (*7*). Both fabrics indicate that the calcite contains Mg in excess of 1000 ppm (*8*). Fabric (A) consists of close-packed bundles of translucent, elongated columnar, fascicular-optic calcite crystals while fabric (B) is characterized by secondary porosity (greenish arrow) where crystal tips show signs of dissolution followed by reprecipitation of single columnar calcite (red circles). This replacement is associated with more variable hydrology, explaining also the presence of impurities (yellow arrows). Since this early diagenesis occurred prior to, or simultaneous to the deposition of the subsequent layer of fascicular optic bundles, the process has not altered (significantly) the chemical properties of the crystals, and in particular the $\delta^{18}O$ signal, which is believed to reflect that of the parent water. Similar fascicular-optic fabrics have also been observed in cryogenic carbonates (9; 10; 11), which could be associated with microbial oxidation of sulfides present in the host-rock. Microbial oxidation would have likely enhanced dissolution of the bedrock subglacially at low temperature (12) and generated most of the HCO_3^- by the dissolution of the (marine) carbonate rocks, as indicated by the overall positive $\delta^{13}C$ values. Scale bar: 1 mm.

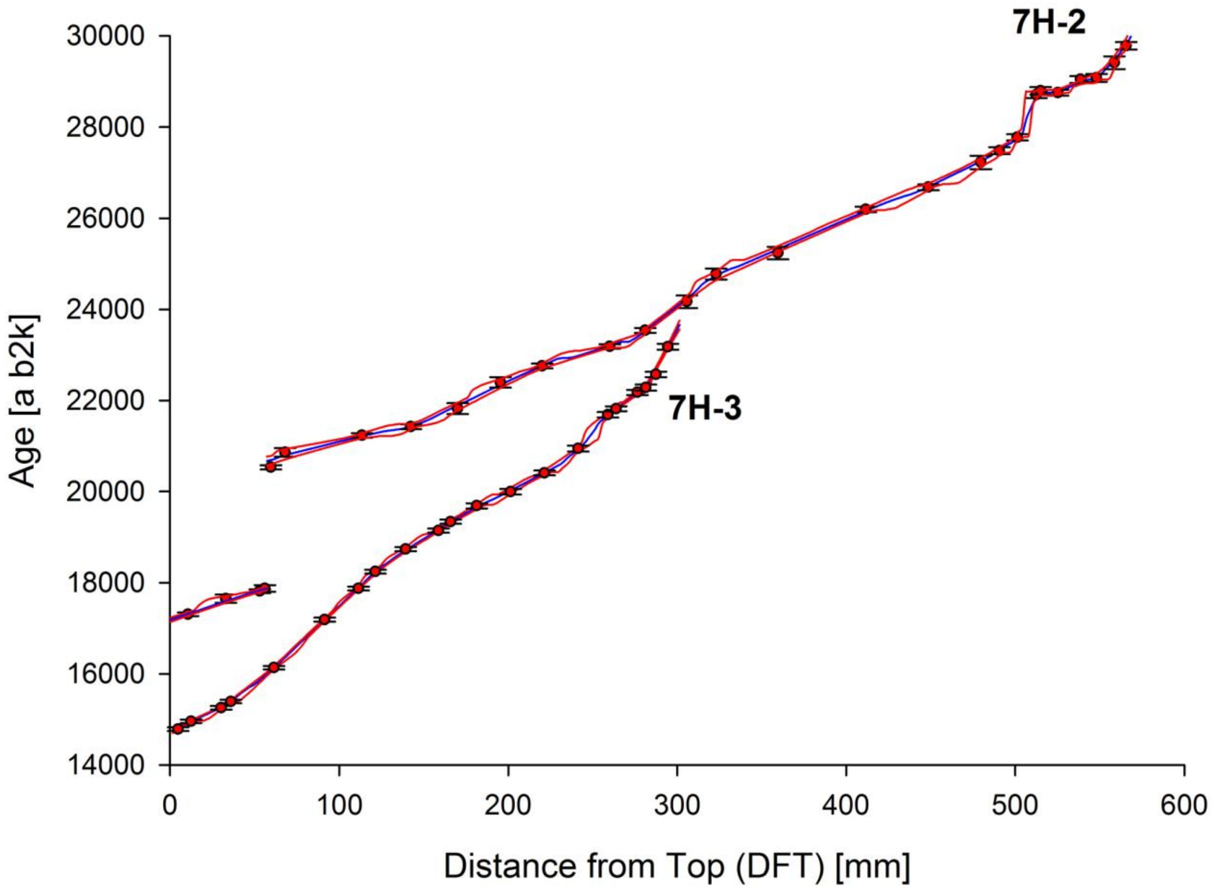

Supplementary Figure 4 Age model of 7H-2 and 7H-3 stalagmites

Age modelling (StalAge; *6*) reveals rather regular growth rates of 40 to 70 µm a^{-1} on average. Coeval deposition is observed between 23.4 and 20.6 ka as well as between 17.9 and 17.3 ka. A major hiatus identified in 7H-2 between 20.5 and 17.8 ka is supported by petrographical changes showing a shift in the growth axis. In contrast, the absence of clear petrographic evidence for a proper growth stop between 28.7 and 27.8 ka (i.e. during Stadial-4) rather suggests that this period was associated with slow deposition. Vertical error bars show the 2σ analytical uncertainty on the U/Th ages.

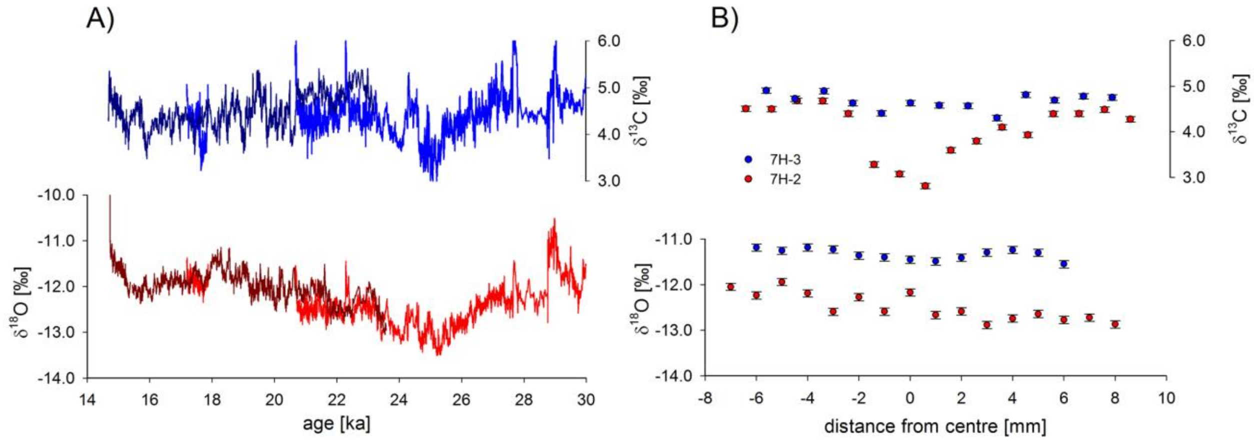

Supplementary Figure 5 Stable isotope measurements on Sieben Hengste speleothems

A) δ^{18}O (red) and δ^{13}C (blue) time series of 7H-2 (bright) and 7H-3 (dark coloured) speleothems. In the overlapping segments, reproducibility is observed between both stalagmites within ±0.6‰ and ±1.0‰ (2σ) for δ^{18}O and δ^{13}C, respectively. A singular event observed on the 7H-2 δ^{18}O record between 22.4 and 22.1 ka is, however, not reproduced, suggesting contrasted hydrological responses. This hydrological control is further supported by a significant correlation (r^2=0.54) between δ^{13}C and δ^{18}O in 7H-2 which points to a slightly more responsive drip than 7H-3. B) δ^{18}O and δ^{13}C analyses along individual growth layers (i.e. Hendy Tests) of 7H-2 and 7H-3. Vertical error bars show the analytical uncertainty (1σ) on stable isotope data. The more positive values observed in 7H-3 suggest that kinetic fractionation affects this sample more strongly. This result is consistent with a somewhat lower drip discharge as inferred from the speleothem growth rate. Overall, the elevated δ^{13}C values reflect the isotopic composition of the host rock and strongly support the lack of soil-derived organic carbon in the karst system. The higher δ^{13}C values observed in 7H-3 suggest an early CO_2 degassing associated with a low drip rate.

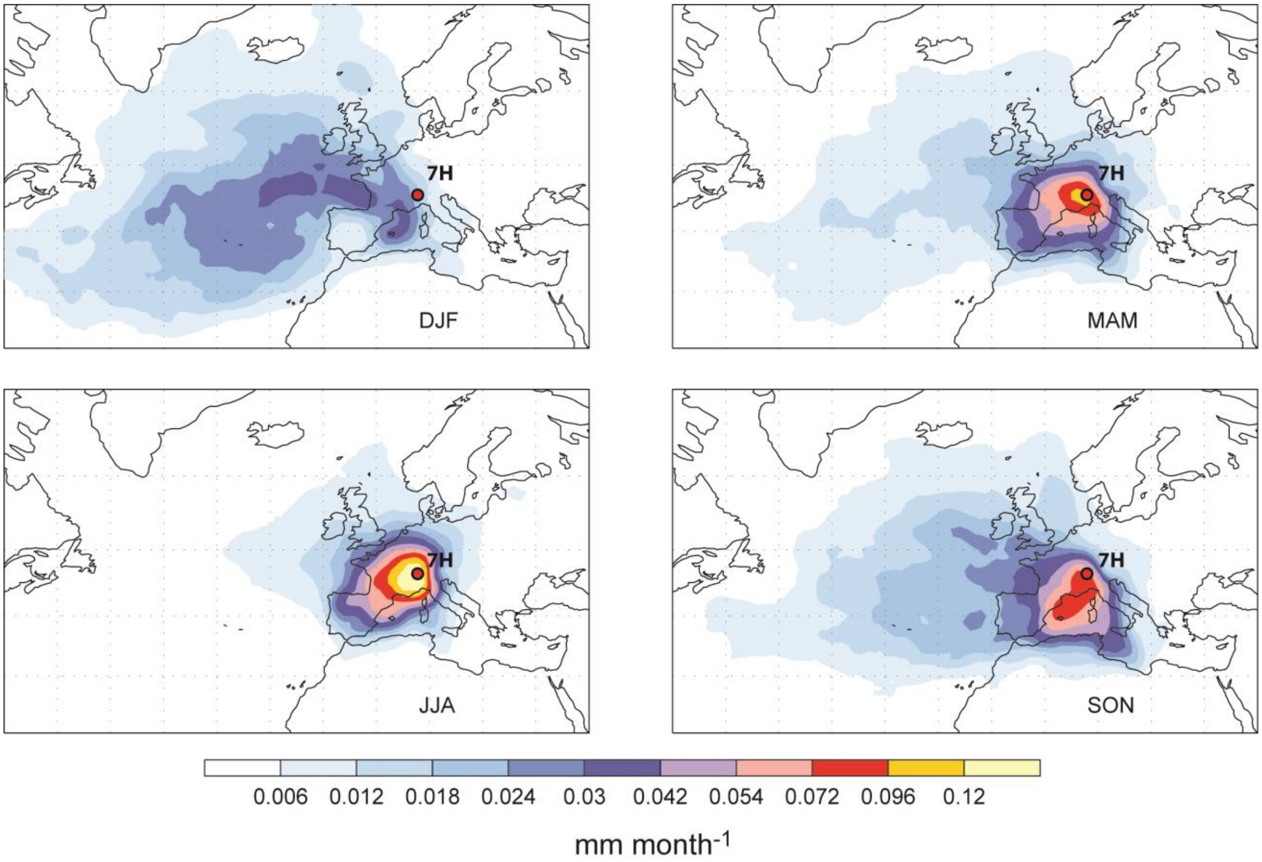

Supplementary Figure 6 Seasonal mean moisture sources of precipitation reaching the Sieben Hengste

Moisture sources from 1995 to 2005 are expressed as evaporation contribution to total meteoric precipitation at the Sieben Hengste (7H; red dot), in mm $month^{-1}$.

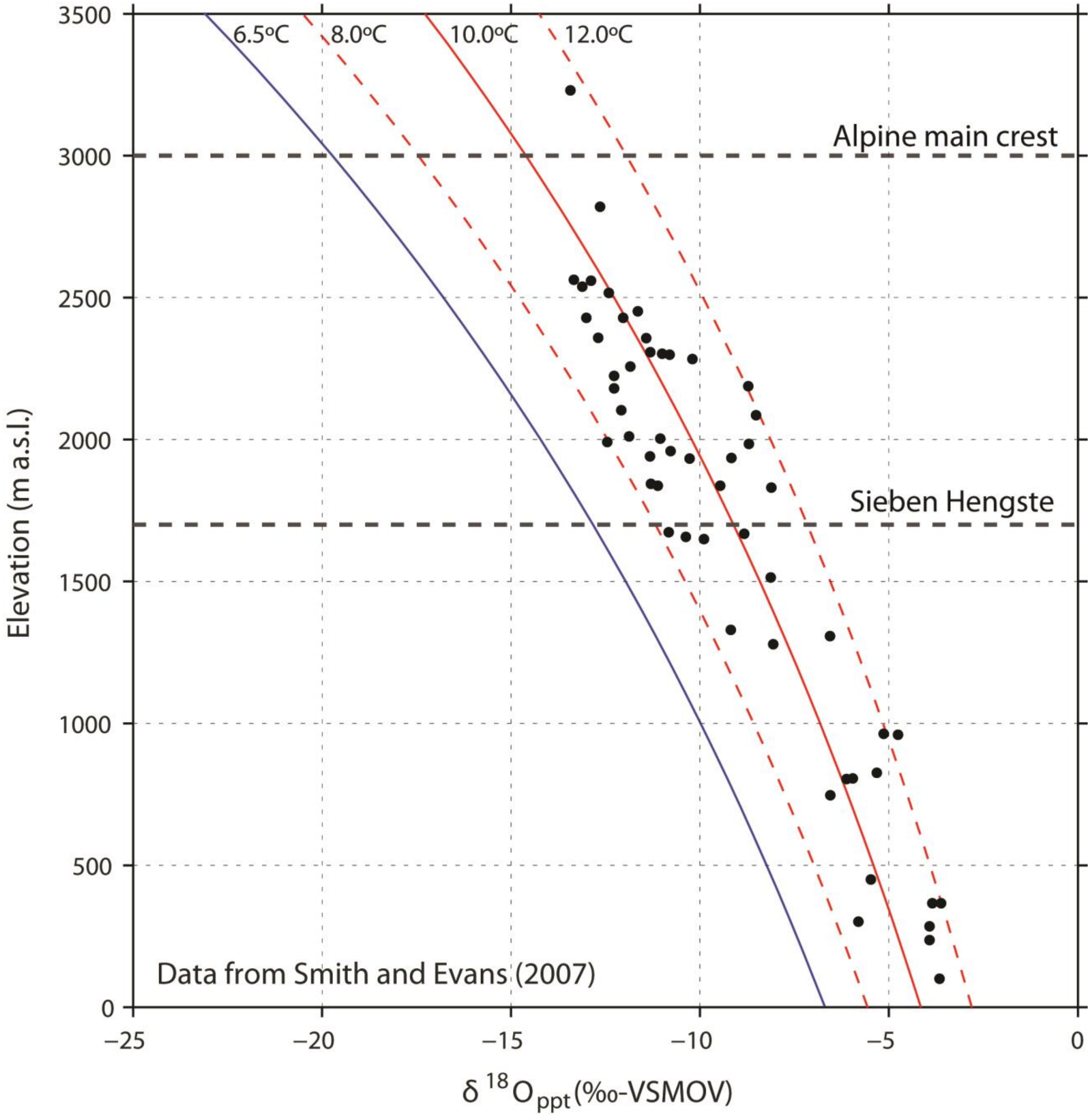

Supplementary Figure 7 Isotopic depletion in ^{18}O versus barrier elevation

Solid curves are from a Rayleigh fractionation model showing the isotopic composition of precipitation from moisture starting a pseudoadiabatic ascent from a surface temperature of 6.5, 8.0, 10.0 and 12.0°C and a vapour isotope value of -11‰ for $\delta^{18}O$. The presented data (*13*) are best explained by a surface temperature range of 8--12ºC (red lines). For a Rayleigh distillation with a source temperature of 6.5ºC (blue line) a two-member mixing model for the isotopic composition at 7H has been constructed. Elevations of 7H and Alpine main crest are indicated by the horizontal dashed line.

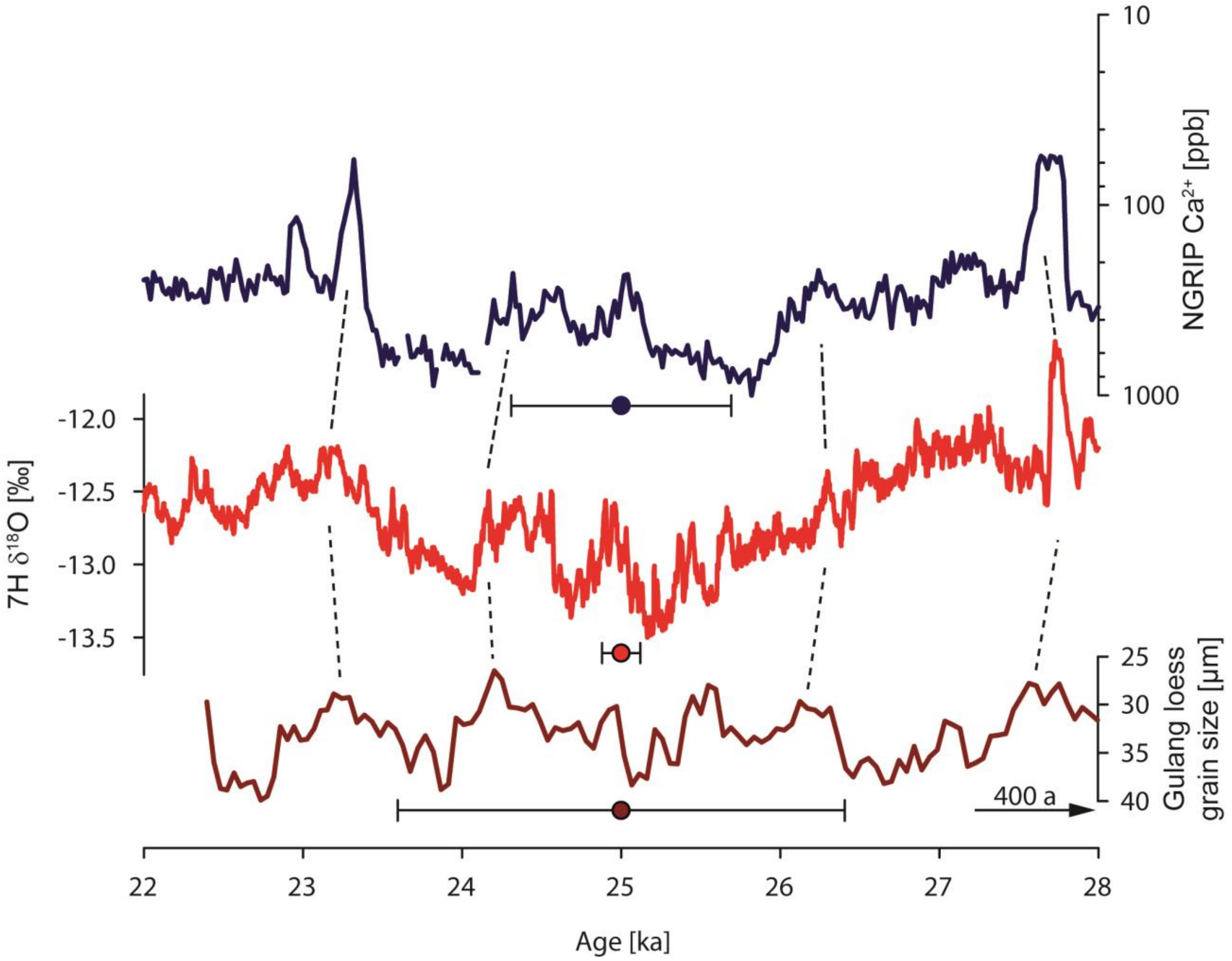

Supplementary Figure 8 Comparison between 7H and the dust record

The composite 7H δ^{18}O record (centre), the Greenland dust record (*14*; top) and the Gulang loess record (*15*; bottom) during the Last Glacial Maximum are shown together with their typical age uncertainties (error bars); NGRIP: maximum counting error (2σ; *16*); 7H: U/Th age (2σ); Gulang: OSL age (1σ). The time axis for the Gulang loess record has been offset by 400 a towards older ages to facilitate comparison with the other time series.

	Mg (m25)	S (m34)	Sr (m88)	Ba (m138)	U (m238)
	ppm	ppm	ppm	ppm	ppm
average	7827	460	871	37	1.069
stand. dev.	782	71	163	9	0.323

Supplementary Table 1 LA-ICPMS data measured on the 7H-2 sample

The measured trace elements all derive from the dissolution of the marine Schrattenkalk carbonates. The elevated magnesium concentration is consistent with the speleothem petrography, suggesting slow but regular drip rates. The presence of sulphur results from the oxidation of pyrite disseminated in the host rock. This interpretation is consistent with secondary gypsum deposits ($\delta^{34}S$: -32 to -26.3 ‰; *17*), which represent a common feature in the Sieben Hengste cave system. Because sulfide oxidation enhances the dissolution capacity of seepage water, it also represents a major process for speleothem deposition in periglacial karst areas (*18*). It is noteworthy that Y, an element which is commonly associated with colloidal transport from the soil, is largely below detection limit (~13 ppb). No chemical laminations were observed suggesting regular drip chemistry associated with a well-mixed aquifer.

Sample	DFT (mm) isotrack	Lab ID	^{238}U (ppb)	^{232}Th (ppt)	^{230}Th / ^{232}Th (atomic x10^{-6})	$\delta^{234}U$* (measured)	^{230}Th / ^{238}U (activity)	^{230}Th Age (a) (uncorrected)	$\delta^{234}U_{Initial}$** (corrected)	^{230}Th Age (a b2K)*** (corrected)
7H2- 8	10.6 ± 2.0	RB185	1383.4 ± 1.3	153 ± 4	34880 ± 810	571.0 ± 1.7	0.2334 ±0.0004	17374 ±38	599.7 ± 1.8	**17304 ±38**
7H2- 31	33.0 ± 2.0	RB97	1291.8 ± 2.2	304 ± 8	17360 ± 480	640.8 ± 2.5	0.2480 ±0.0010	17662 ±86	673.6 ± 2.6	**17649 ±86**
7H2- 45	53.0 ± 2.0	RB186	1170.5 ± 1.2	110 ± 3	43680 ± 1080	636.0 ± 1.9	0.2500 ±0.0010	17884 ±43	668.9 ± 2.0	**17814 ±43**
7H2- 58	56.0 ± 2.0	top	1246.8 ± 2.6	286 ±32	17668 ± 1969	612.5 ±2.2	0.2462 ±0.0009	17884 ±74	644.2 ± 2.3	**17872 ±74**
7H2- 51	59.6 ± 2.0	RB187	1361.9 ± 1.4	652 ± 13	8060 ± 160	348.6 ± 1.7	0.2340 ±0.0004	20609 ±46	369.4 ± 1.8	**20531 ±47**
7H2- 65	68.0 ± 2.0	RB98	1518.4 ± 2.7	355 ± 7	16690 ± 310	347.9 ± 2.3	0.2370 ±0.0010	20870 ±96	369.0 ± 2.5	**20856 ±96**
7H2- 109	113.4 ± 2.0	RB188	1416.8 ± 1.6	127 ± 3	44640 ± 1060	356.5 ± 1.8	0.2430 ±0.0004	21302 ±50	378.6 ± 1.9	**21232 ±50**
7H2- 140	142.4 ± 2.0	RB205	1537.7 ± 1.8	92 ± 3	67070 ± 1870	355.0 ± 1.8	0.2440 ±0.0010	21491 ±54	377.2 ± 1.9	**21422 ±54**
7H2- 167	170.0 ± 2.0	RB123	1489.9 ± 2.4	29 ± 7	208070 ± 51930	361.6 ± 2.6	0.2490 ±0.0010	21830 ±122	384.6 ± 2.8	**21821 ±122**
7H2- 192	195.4 ± 2.0	RB99	1382.2 ± 2.8	146 ± 8	40190 ± 2140	376.3 ± 2.8	0.2580 ±0.0010	22410 ±114	400.9 ± 3.0	**22399 ±114**
7H2- 220	220.0 ± 2.0	RB206	1763.2 ± 1.9	82 ± 2	96010 ± 2900	423.6 ± 1.7	0.2710 ±0.0010	22828 ±52	451.8 ± 1.8	**22759 ±52**
7H2- 254	260.0 ± 2.0	RB189	1420.0 ± 1.4	73 ± 2	88050 ± 2580	412.3 ± 1.7	0.2740 ±0.0004	23257 ±51	440.3 ± 1.8	**23188 ±51**
7H2- 277	281.0 ± 2.0	RB242	1373.0 ± 1.4	56 ± 2	110820 ± 3390	401.9 ± 1.8	0.2750 ±0.0004	23609 ±53	429.6 ± 1.9	**23541 ±53**
7H2- 300	305.6 ± 2.0	RB124	1026.4 ± 1.5	131 ± 7	35900 ± 2020	385.6 ± 2.6	0.2780 ±0.0010	24182 ±139	412.8 ± 2.8	**24170 ±139**
7H2- 319	323.0 ± 2.0	RB100	1415.8 ± 2.6	278 ± 8	24900 ± 720	444.9 ± 2.5	0.2970 ±0.0010	24790 ±123	477.1 ± 2.7	**24777 ±123**
7H2- 354	359.6 ± 2.0	RB125	921.6 ± 1.4	208 ± 7	23420 ± 760	530.5 ± 2.9	0.3200 ±0.0010	25251 ±135	569.6 ± 3.1	**25238 ±135**
7H2- 407	411.6 ± 2.0	RB190	1032.2 ± 1.0	480 ± 10	11180 ± 230	457.9 ± 1.7	0.3160 ±0.0010	26272 ±58	493.1 ± 1.9	**26195 ±59**
7H2- 443	448.6 ± 2.0	RB240	1365.2 ± 1.3	267 ± 6	26470 ± 550	424.7 ± 1.7	0.3130 ±0.0010	26753 ±60	458.0 ± 1.8	**26681 ±60**
7H2- 474	479.6 ± 2.0	RB126	1011.2 ± 1.6	391 ± 7	14440 ± 270	512.5 ± 2.9	0.3390 ±0.0010	27244 ±146	553.5 ± 3.1	**27227 ±146**
7H2- 491	490.6 ± 2.0	RB308	1149.7 ± 1.7	766 ± 15	8580 ± 170	533.5 ± 2.0	0.3470 ±0.0001	27564 ±76	576.7 ± 2.2	**27483 ±77**
7H2- 500	501.2 ± 2.0	RB241	695.8 ± 0.5	735 ± 15	5590 ± 110	564.7 ± 1.9	0.3580 ±0.0010	27865 ±67	610.9 ± 2.1	**27778 ±68**
7H2- 512	512.4 ± 2.0	RB416	728.4 ± 0.7	481 ± 10	8839 ± 178	508.4 ± 2.2	0.3541 ±0.0006	28733 ±71	551.3 ± 2.4	**28710 ±71**
7H2- 515	515.0 ± 2.0	RB309	1058.1 ± 1.6	284 ± 6	20430 ± 420	413.3 ± 1.9	0.3330 ±0.0010	28871 ±81	448.3 ± 2.0	**28796 ±81**
7H2- 523	525.2 ± 2.0	RB207	1415.3 ± 1.4	164 ± 4	46370 ± 1050	390.0 ± 1.7	0.3270 ±0.0010	28828 ±67	423.1 ± 1.8	**28757 ±67**
7H2- 539	538.6 ± 2.0	RB417	1519.2 ± 1.8	255 ± 5	32745 ± 679	410.8 ± 1.9	0.3339 ±0.0006	29062 ±74	446.0 ± 2.1	**29049 ±74**
7H2- 548	548.0 ± 2.0	RB311	1483.8 ± 2.4	530 ± 11	15490 ± 310	414.2 ± 2.0	0.3360 ±0.0010	29157 ±86	449.7 ± 2.2	**29081 ±86**
7H2- 556	558.6 ± 2.0	RB101	1302.2 ± 2.3	313 ± 7	23620 ± 540	436.8 ± 2.7	0.3440 ±0.0010	29430 ±139	474.6 ± 2.9	**29416 ±139**
7H2- 563	565.6 ± 2.0	RB208	646.2 ± 0.6	1555 ± 31	2520 ± 50	510.4 ± 1.7	0.3670 ±0.0010	29899 ±74	555.3 ± 1.9	**29786 ±81**
7H2- 566	568.0 ± 2.0	base	555.9 ± 1.0	2203 ± 35	1545 ± 25	478.9 ± 2.0	0.3715 ±0.0013	31042 ±135	522.6 ± 2.2	**30958 ±145**
7H3- 3	3.4 ± 2.0	RB439	927.5 ± 1.1	107 ± 3	29332 ± 789	609.4 ± 2.3	0.20517 ±0.00044	14748 ±40	635.2 ± 2.4	**14735 ±40**
7H3- 12	11.0 ± 2.0	RB300	996.0 ± 1.3	272 ± 6	12577 ± 263	617.5 ± 2.1	0.20852 ±0.00042	14922 ±38	644.1 ± 2.2	**14907 ±38**
7H3- 29	28.8 ± 2.0	RB425	1126.7 ± 1.3	68 ± 2	57988 ± 1619	624.3 ± 2.3	0.21329 ±0.00043	15217 ±40	651.7 ± 2.5	**15205 ±40**
7H3- 35	34.6 ± 2.0	RB452	962.4 ± 1.1	231 ± 5	14724 ± 307	615.5 ± 2.3	0.21395 ±0.00042	15356 ±40	642.8 ± 2.5	**15341 ±40**
7H3- 60	60.0 ± 2.0	RB440	904.8 ± 0.9	107 ± 2	31184 ± 712	621.7 ± 2.1	0.22447 ±0.00041	16096 ±39	650.5 ± 2.2	**16083 ±39**
7H3- 90	90.0 ± 2.0	RB426	1236.3 ± 1.4	94 ± 2	50674 ± 1295	588.0 ± 2.2	0.23316 ±0.00042	17150 ±42	617.2 ± 2.3	**17138 ±42**
7H3- 110	110.0 ± 2.0	RB482	1025.0 ± 1.1	167 ± 1	24604 ± 200	593.8 ± 2.1	0.24272 ±0.00043	17836 ±43	624.5 ± 2.2	**17822 ±43**
7H3- 120	120.0 ± 2.0	RB427	1214.9 ± 1.4	39 ± 1	124142 ± 4740	560.5 ± 2.1	0.24210 ±0.00043	18203 ±44	590.0 ± 2.2	**18191 ±44**
7H3- 138	138.0 ± 2.0	RB483	1170.5 ± 1.3	449 ± 2	10139 ± 39	484.6 ± 2.0	0.23600 ±0.00042	18704 ±46	510.8 ± 2.2	**18686 ±46**
7H3- 157	157.4 ± 2.0	RB441	1243.9 ± 1.4	65 ± 2	78017 ± 2063	526.4 ± 2.0	0.24755 ±0.00041	19103 ±44	555.6 ± 2.1	**19091 ±44**
7H3- 165	164.6 ± 2.0	RB428	1370.9 ± 1.7	23 ± 1	241063 ± 13224	499.0 ± 2.1	0.24532 ±0.00045	19299 ±48	526.9 ± 2.2	**19287 ±48**
7H3- 180	180.0 ± 2.0	RB484	1446.9 ± 2.0	104 ± 1	51394 ± 710	353.3 ± 2.3	0.22474 ±0.00047	19649 ±58	373.5 ± 2.4	**19636 ±58**
7H3- 200	200.0 ± 2.0	RB442	1749.6 ± 2.4	55 ± 2	120631 ± 3758	353.2 ± 1.9	0.22796 ±0.00057	19958 ±63	373.6 ± 2.1	**19946 ±63**
7H3- 220	220.0 ± 2.0	RB429	1712.7 ± 2.2	150 ± 3	43728 ± 996	356.4 ± 1.9	0.23285 ±0.00044	20370 ±52	377.5 ± 2.0	**20357 ±52**
7H3- 240	240.0 ± 2.0	RB485	1374.8 ± 2.0	485 ± 2	11299 ± 47	373.4 ± 2.2	0.24156 ±0.00056	20911 ±65	396.1 ± 2.4	**20892 ±65**
7H3- 258	257.6 ± 2.0	RB443	1645.3 ± 2.2	136 ± 3	50127 ± 1131	382.3 ± 2.0	0.25096 ±0.00048	21648 ±57	406.4 ± 2.1	**21635 ±57**
7H3- 262	262.4 ± 2.0	RB430	1372.4 ± 1.6	42 ± 2	136354 ± 5569	383.9 ± 2.0	0.25263 ±0.00045	21778 ±55	408.2 ± 2.1	**21766 ±55**
7H3- 275	275.0 ± 2.0	RB266	1509.7 ± 2.0	286 ± 6	22302 ± 465	381.9 ± 1.8	0.25612 ±0.00049	22143 ±57	406.5 ± 1.9	**22129 ±57**
7H3- 280	280.0 ± 2.0	RB486	1451.6 ± 2.2	108 ± 2	57485 ± 934	397.6 ± 2.3	0.26020 ±0.00057	22247 ±67	423.3 ± 2.5	**22235 ±67**
7H3- 286	286.0 ± 2.0	GM315	1066.8 ± 1.0	2260 ± 45	2115 ± 43	439.6 ± 1.7	0.27184 ±0.00044	22580 ±50	468.5 ± 1.8	**22524 ±58**
7H3- 293	293.0 ± 2.0	RB444	798.6 ± 0.9	29 ± 2	127748 ± 7217	439.4 ± 2.3	0.27794 ±0.00053	23142 ±64	469.0 ± 2.4	**23130 ±64**

Supplementary Table 2 $^{230}Th/^{234}U$ ages of 7H-2 and 7H-3 stalagmites

Analytical errors are at 95% confidence level.

*$\delta^{234}U = ([^{234}U/^{238}U]_{activity} - 1) \times 1000$.

** $\delta^{234}U_{initial}$ was calculated based on ^{230}Th age (T), i.e., $\delta^{234}U_{initial} = \delta^{234}U_{measured} \times e^{\lambda 234 \times T}$.

Following decay constants were used: $\lambda^{230} = 9.158 \times 10^{-6}\ a^{-1}$ (19); $\lambda^{234} = 2.826 \times 10^{-6}\ a^{-1}$ (19); $\lambda^{238} = 1.551 \times 10^{-10}\ a^{-1}$ (20). Corrected ^{230}Th ages assume an initial $^{230}Th/^{232}Th$ atomic ratio of $4.4 \pm 2.2 \times 10^{-6}$; i.e. values in secular equilibrium with a bulk Earth $^{232}Th/^{238}U$ value of 3.8. The errors are arbitrarily assumed to be 50%.

*** a b2k stands for before 2000 AD.

Supplementary Discussion

The modern annual precipitation at the study site averages 2000 mm, nearly 40% of which fall as snow between November and April. The $\delta^{18}O$ of precipitation typically averages -13.1±0.6 ‰-VSMOW with a seasonal amplitude of ca. 10 ‰ (*21*). 10-day backward trajectories calculated from an ERA-Interim reanalysis data for the years 1995-2005 were started at 6 hours intervals on a 60x60 km horizontal grid with 30 hPa vertical intervals. A quantitative Lagrangian moisture source diagnostic (*22, 23*) reveals a dominant transport direction from the West, which is particularly pronounced during the winter season (Supplementary Figure 6). However, a seasonally varying fraction of moisture ranging on average between 10 and 30 % also reaches the 7H site from the South. Our method underestimates the seasonal variability since convective (summer) precipitation is less reliably captured. From climatological studies based on precipitation measurements, it is known that about 1/3 of the precipitation along the Northern Alps falls during the summer season, with the remaining 2/3 distributed equally over the other seasons (24).

While Mediterranean moisture sources can contribute substantial amounts of precipitation to the southern Alps during spring and autumn, cluster analyses of extreme rainfall events in northwestern Italy suggest that the strongest precipitation events are likely associated with significant moisture advection from the North Atlantic during periods of intense northward flow to the southern Alpine range (*25*). An increasing number of studies provide evidence that the North Atlantic is the dominant oceanic moisture source of alpine precipitation also in periods of strong southerly moisture advection (*26*). Considering drying ratios as low as 35 % (*27*) a substantial amount of North Atlantic sourced moisture may eventually reach the Sieben Hengste, also with southerly transport routes.

The ^{18}O depletion associated with orographic precipitation depends predominantly on thermodynamic effects (*28*) with topography being the dominant control on the isotope ratio of precipitation. Empirical observations from the Andes (*13*) confirm that $\delta^{18}O$ values largely follow a Rayleigh fractionation model with a mean gradient of ca. 3-4 ‰ per 1000 m barrier elevation (Supplementary Figure 7). Considering all other parameters equal and assuming an average topographic barrier south of the study site at ca. 3000 m a.s.l., the potential difference in the $\delta^{18}O$ signal associated with southerly advection reaches 5 to 7 ‰ as compared to the direct route from the North-West. Using the Rayleigh fractionation model a two end-member mixing model has been constructed for different surface temperatures. Given the approximate 50% threshold value at 16.3‰ $\delta^{18}O$-VSMOV (Figure 4), a best matching mixing model was obtained with end-member values of 19.7‰ for the Alpine main crest and 12.8‰ for the direct approach to 7H, based on an initial surface temperature of 6.5ºC. This transfers the $\delta^{18}O$ signal of between 1.5 and 3 ‰ as inferred from the peak glacial $\delta^{18}O$ shift in the 7H-record to a southern humidity fraction ranging from 25 to 65 % of the average annual precipitation at Sieben Hengste during the LGM. Sensitivity of the estimate is about 10% less southerly contribution per degree in surface temperature change.

In contrast to the storm trajectories, local temperature effects are assumed to play only a second order role in the isotope fractionation affecting meteoric precipitation reaching the Sieben Hengste. Changing the temperature of condensation is expected to produce comparatively small amplitudes in isotope fractionation on palaeoclimate timescales. Typically, major precipitation events go along with substantial advection of warm moist air, which, due to its transient nature, is hardly reflected in the mean annual air temperature record. For example, temperature of precipitation in Greenland is up to 15 K warmer than the annual mean temperatures (22).

From present-day synoptic analysis it is well known that events of heavy precipitation in the Southern Alps are typically associated with intense moisture advection from the North Atlantic, in some cases tapping into the subtropical pool of high humidity (*25*). Such cases of meridional moisture advection are typically associated with a trough signature at mid-tropospheric levels, also known as narrow potential vorticity streamers. The jet stream bends around the outside of these troughs (streamers), and induces the dominant transport pattern in the troposphere below. When the Fennoscandian ice cap was present during the LGM, atmospheric motion was different as compared to today, (i) by a equatorward shift of the sea-ice margin, and thus the zone for baroclinic storm development, and (ii) by inducing a frequent cold high-pressure residing over the ice cap, blocking the flow and deflecting the jet stream, inducing pronounced undulations, known as Rossby wave breaking. With the tropics (and subtropics) cooling less

than high-latitudes during the LGM, substantial moisture should have been available in proximity to the equatorward shifted baroclinic zone (storm track) and the overlying jet stream. As the disturbances due to the blocking anticyclone not just die off, but can continue to influence weather downstream, it is possible that additional wave breaking was induced over central and eastern Asia, triggering again strong meridional advection patterns underneath upper-level jet excursions. This time the systems would not transport moisture towards the Alps, but generate storms that could transport large amounts of dust into higher (Arctic) latitudes. Additional or alternative mechanisms may have included (i) increased deflation of aeolian material in the dust sources, (ii) increased wind speed due to changed land cover, (iii) larger deserts, (iv) less effective removal processes, (v) a more direct transport pathway towards the Arctic.

Supplementary References

1. Häuselmann, P., Jeannin, P.-Y. Sieben Hengste, Switzerland. In Gunn (ed.), Encyclopedia of Caves and Karst Science, Fitzroy Dearborn, 647-649 (2004).
2. Pfiffner, O.A. Structural Map of the Helvetic Zone of the Swiss Alps, including Vorarlberg (Austria) and Haute Savoie (France), 1:100 000.*Geological Special Map*, **128**. Explanatory notes (2011).
3. Ziegler, M.A. A study of the Lower Cretaceous facies developments in the Helvetic Border Chain, north of the Lake of Thun (Switzerland). *Eclog. Geologic. Helvet.,* **60**, 509-527 (1967).
4. Bini, A., Buoncristiani, F., Couterrand, S., Ellwanger, D., Fleber, M., Florineth, D., Graf, H.R., Keller, O., Kelly, M., Schlüchter, C., Schoeneich, P. Switzerland during the Last Glacial Maximum (LGM) 1:500000. Federal Office of Topography Swisstopo, Switzerland. (2009).
5. Miorandi, R., Borsato, A., Frisia, S., Fairchild, I.J., Richter, D.K. Epikarst hydrology and implications for stalagmite capture of climate changes at Grotta di Ernesto (NE Italy): results from long-term monitoring. *Hydrol. Process.*, **24**, 3101-3114 (2010).
6. Scholz, D., Hoffmann, D.L. StalAge An algorithm designed for construction of speleothem age models. *Quat. Geochronology.*, **6**, 369-382 (2011).
7. Kendall, A.C. Fascicular-optic calcite – replacement of bundled acicular carbonate cements. *J. Sedim. Petrol.*, **47**, 1056-1062 (1977).
8. Frisia, S., Borsato, A.. Karst. In A.M. Alonso-Zarza and L.H. Tanner (eds), Carbonates in Continental Settings: Facies, Environments, and Processes Developments in Sedimentology. Elsevier, 269–318 (2010).
9. Richter, D.K., Riechelmann, D.F.Ch. Late Pleistocene cryogenic calcite spherolites from the Malachitdom Cave (NE Rhenish Slate, Mountains, Germany): origin, unusual internal structure and stable C-O isotope composition, *Int. J. Speleology*, **37**, 119-129 (2008).
10. Žák, K., Onac, B., Perşoiu, A. Cryogenic carbonates in cave environments: A review. *Quat.Int.*, **187**, 84-96 (2008).
11. Luetscher, M., Borreguero, M., Moseley, G., Spötl, C., Edwards, L.E.. Alpine permafrost thawing during the Medieval Warm Period identified from cryogenic cave carbonates. *The Cryosphere*, **7**, 1073-1081 (2013).
12. Mitchell, A.C., Brown, G.H., Modeling geochemical and biogeochemical reactions in subglacial environments. *Arct. Antarct. Alp. Res.*, **40**, 531-547 (2008).
13. Smith, R.B., Evans, J.P. Orographic precipitation and water vapour fractionation over the Southern Andes. *J. Hydrometeorology*, **8**, 3-19 (2007).
14. Bigler, M. Hochauflösende Spurenstoffmessungen an polaren Eisbohrkernen: Glaziochemische und klimatische Prozessstudien. PhD, University of Bern, Switzerland, 148 p. (2004).
15. Sun, Y., Clemens, S.C., Morrill, C., Lin, X., Wang, X., An, Z., Influence of Atlantic meridional overturning circulation on the East Asian winter monsoon. *Nature Geosci.* **5**, 46-49 (2012).
16. Andersen, K.K., Svensson, A., Johnsen, S.J., Rasmussen, S.O., Bigler, M., Röthlisberger, R., Ruth, U., Siggaard-Andersen, M.L. Steffensen, J.P., Dahl-Jensen, D., Vinther, B.M., Clausen, H.B. The Greenland ice core chronology 2005, 15-42 ka. Part 1: constructing the time scale. *Quat. Sci. Rev.*, **25**, 3246-3257 (2006).

17. Filipponi, M. Spatial Analysis of Karst Conduit Networks and Determination of Parameters Controlling the Speleogenesis along Preferential Lithostratigraphic Horizons. PhD Thesis, Ecole Polytechnique Fédérale de Lausanne.

18. Luetscher, M., Hoffmann, D., Frisia, S., Spötl, C. Holocene glacier history from alpine speleothems, Milchbach cave, Switzerland. *Earth Plan. Sci. Lett.*, **302**, 95–106 (2011).

19. Cheng, H., Edwards, R.L., Hoff, J., Gallup, C.D., Richards, D.A., Asmerom, Y., 2000. The half-lives of uranium-234 and thorium-230, *Chem. Geol.* **169**, 17-33 (2000).

20. Jaffey, A.H., Flynn, K.F., Glendenin, L.E., Bentley, W.C., Essling, A. M., Precision measurement of half-lives and specific activities of 235U and 238U. *Phys. Rev*. **C4**, 1889–1906 (1971).

21. Schürch, M., Kozel, R., Schotterer, U., Tripet, J.-P. Observation of isotopes in the water cycle -the Swiss National Network (NISOT). *Environm. Geol.*, **45**, 1–11 (2003).

22. Sodemann, H., Schwierz, C.; Wernli, H., 2008. Interannual variability of Greenland winter precipitation sources: Lagrangian moisture diagnostic and North Atlantic Oscillation influence. *J. Geophys. Res. Atm.*, **113**, D3 (2008).

23. Sodemann, H., Zubler, E. Seasonal and inter-annual variability of the moisture sources for Alpine precipitation during 1995-2002. *Int. J. Climatol.*., **30**, 947–961 (2010).

24. Frei, C, Schär, C. A Precipitation climatology of the Alps from high-resolution rain-gauge observations. *Int. J. Climatol.,* **18**: 873–900 (1998).

25. Pinto, J.G., Ulbrich, S., Parodi A., Rudari R., Boni G., Ulbrich U. Identification and ranking of extraordinary rainfall events over Northwest Italy: The role of Atlantic moisture, *J. Geophys. Res. Atmos.*, **118**, 2085–2097, doi:10.1002/jgrd.50179 (2013).

26. Winschall, A., Sodemann, H., Pfahl, S., Wernli, H., How important is intensified evaporation for Mediterranean precipitation extremes? *J. Geophys. Res. Atmos.*, **119**, 5240–5256, doi:10.1002/2013JD021175 (2014).

27. Smith, R.B., Jiang, Q., Fearon, M.G., Tabary, P., Dorninger, M., Doyle, J.D., Benoit, R. Orographic precipitation and air mass transformation: an Alpine example. *Q.J.R. Meteorol. Soc.*, **129**, 433-454 (2003).

28. Stern, L.A., Blisniuk, P.A. Stable isotope composition of precipitation across the southern Patagonian Andes. *J. Geophys. Res. Atm.*, **107**, D23 (2002).
